# Comparative enrichment of complete ammonium oxidation bacteria in floccular sludge reactors: Sequencing batch reactor vs. continuous stirred tank reactor

**DOI:** 10.1016/j.wroa.2025.100305

**Published:** 2025-01-22

**Authors:** Ying Zhu, Jiaying Hou, Fangang Meng, Meiying Xu, Limin Lin, Linyan Yang, Xueming Chen

**Affiliations:** a College of Environment and Safety Engineering, Fuzhou University, Fuzhou 350116, PR China; b Guangdong Provincial Key Laboratory of Environmental Pollution Control and Remediation Technology (Sun Yat-sen University), Guangzhou 510275, PR China; c State Key Laboratory of Applied Microbiology Southern China, Institute of Microbiology, Guangdong Academy of Sciences, Guangzhou 510070, PR China; d School of Engineering, Westlake University, Hangzhou, Zhejiang 310030, PR China; e School of Resources and Environmental Engineering, East China University of Science and Technology, Shanghai 200237, PR China

**Keywords:** Ammonium-oxidizing bacteria (AOB), Complete ammonium oxidation (comammox) bacteria, Continuous stirred tank reactor (CSTR), r/K-strategists, Sequencing batch reactor (SBR)

## Abstract

This study attempted to compare the enrichment of complete ammonium oxidation (comammox) bacteria, which are affiliated with *Nitrospira* and not able to generate nitrous oxide (N_2_O, a potent greenhouse gas) through biological pathways, in two commonly-utilized configurations of floccular sludge reactors, i.e., sequencing batch reactor (SBR) and continuous stirred tank reactor (CSTR), under the ammonium condition of mainstream wastewater (i.e., 40.0 g-N/m^3^). The results in terms of nitrification performance and microbial analyses during 216-d operation showed that compared with SBR offering a fluctuating but generally higher *in-situ* ammonium concentration (i.e., 1.0–6.0 g-N/m^3^) which was favorable for the growth of ammonium-oxidizing bacteria (AOB, belonging to *Nitrosomonas* in this study), CSTR managed to lower the *in-situ* ammonium level to < 2.0 g-N/m^3^, thus creating a competitive advantage for comammox bacteria with a highly oligotrophic lifestyle. Such an argument was further supported by dedicated batch tests which revealed that *Nitrospira*-dominant sludge had a lower maximum ammonium oxidation rate and lower apparent ammonium and oxygen affinity constants than *Nitrosomonas*-dominant sludge (i.e., 33.5 ± 2.1 mg-N/h/g-MLVSS vs. 139.9 ± 26.7 mg-N/h/g-MLVSS, 1.1 ± 0.1 g-N/m^3^ vs. 17.6 ± 4.6 g-N/m^3^, and 0.017 ± 0.002 g-O_2_/m^3^ vs. 0.037 ± 0.013 g-O_2_/m^3^, respectively), proving the nature of comammox bacteria as a K-strategist. Overall, this study not only provided useful insights into the effective enrichment of comammox bacteria in floccular sludge but also further revealed the interactions between comammox bacteria and AOB, thereby contributing to the future development of comammox-inclusive biological nitrogen removal technologies for sustainable wastewater treatment.

## Highlights

• Efficacy of SBR and CSTR in enriching comammox bacteria was compared.
• Comammox bacteria were more suited to grow in CSTR with lower *in-situ* NH_4_^+^.
• Identification of K_NH4__+_, K_O2_, and V for enriched *Nitrospira*-dominant sludge.

## Graphical abstract

**Figure:**
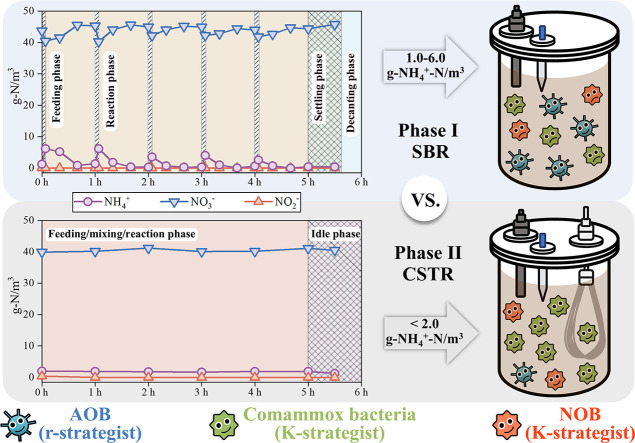

## 1 Introduction

Nitrification, a key process in the biogeochemical nitrogen cycle, plays a significant role in achieving biological nitrogen removal (BNR) at wastewater treatment plants (WWTPs) (Yu et al., 2018). Traditionally, nitrification has been known to comprise two distinct biological steps, including ammonium oxidation dominated by ammonium-oxidizing archaea (AOA) or bacteria (AOB) and nitrite oxidation catalyzed by nitrite-oxidizing bacteria (NOB). The discovery of complete ammonium oxidation (comammox) (Daims et al. 2015; van Kessel et al. 2015) refreshed our understanding of nitrification.

Comammox bacteria, belonging to *Nitrospira* sublineage II, possess complete nitrification genes, including ammonia monooxygenase (AMO), hydroxylamine dehydrogenase (HAO), and nitrite oxidoreductase (NXR), thus directly enabling the oxidation of ammonium via nitrite to nitrate (Zhu et al. 2022). Compared with the traditional nitrification process that involves the cooperation between AOB and NOB, the truncated nitrification carried out by comammox bacteria can reduce the metabolic cost for the cell (Koch et al. 2018). Moreover, unlike AOB, comammox bacteria fix carbon dioxide (CO_2_) through the reductive tricarboxylic acid cycle with low oxygen demand (Shi et al. 2023). At the same time, the intrinsic lack of nitric oxide oxidoreductase (NOR) prevents comammox bacteria from generating nitrous oxide (N_2_O) through biological pathways (Han et al. 2021; Kits et al. 2019). These distinctive traits of comammox bacteria offer new opportunities to achieve BNR with low energy consumption and N_2_O production, leading to a growing interest in enriching comammox bacteria to allow in-depth research.

Comammox bacteria have high affinities for substrates (Koch et al. 2018), slow growth (Vilardi et al. 2023), and the ability to survive in systems featuring a long sludge retention time (SRT) (Hou et al. 2024; Huang et al. 2022). These characteristics make comammox bacteria particularly suitable to thrive in biofilm/granular sludge-based reactors with attached microbial communities. Huang et al. (2022) and Zhao et al. (2022) successfully achieved complete nitrification and selective enrichment of comammox bacteria in biofilm reactors filled with sponge carriers and AnoxK Z-carriers, respectively, and Fujitani et al. (2020) managed to enrich low-abundance comammox bacteria from nitrifying granules in a fixed-bed continuous feeding bioreactor. Up-to-date analyses of the kinetics of comammox bacteria confirmed their preference for oligotrophic environments (Kits et al. 2017; Sakoula et al. 2020), aligning with their documented prevalence in environments with limited ammonium availability, such as soils and drinking water systems (He et al. 2021; Palomo et al. 2022; Wang et al. 2017). Not only that, the presence and activity of comammox bacteria have also been frequently reported at WWTPs mainly adopting floccular sludge-based treatment technologies (Sato et al. 2021; Spasov et al. 2020; Xu et al. 2022; Zheng et al. 2019). Compared with biofilm/granular sludge, the low mass transfer resistance of substrates in floccular sludge would better reflect the competitive relationship between comammox bacteria and other nitrifiers (i.e., AOB and NOB) for common substrates (e.g., ammonium, dissolved oxygen (DO), and nitrite) in such an environment where substrate concentrations are approximately homogeneous. Competition between these nitrifiers may lead to the formation of activated sludge dominated by distinctive microorganisms in different reactors under varying operating conditions. However, the extant literature cannot fully elucidate the effects of reactor types and operating conditions on the dominant microorganisms enriched in activated sludge.

Sequencing batch reactor (SBR) whose typical operation consists of feeding, reaction, settling, decanting, and idle steps in a single reactor (Lu et al. 2018) is a commonly used floccular sludge reactor configuration. SBR can be used to treat wastewater with fluctuations in quality, such as domestic wastewater, and stands out for its flexibility in operation and high treatment efficiency (Askari et al. 2024; Kwon et al. 2023). Recent studies have found that SBR could enrich comammox bacteria by prolonging the SRT and maintaining a low DO level in the reactor. Hou et al. (2024) successfully enriched comammox bacteria-containing floccular sludge after > 200 d using a lab-scale SBR at influent ammonium of 40 g-N/m^3^, DO of 0.5 ± 0.1 g-O_2_/m^3^, and an uncontrolled long SRT. However, the efficiency of SBR to successfully enrich comammox bacteria-dominant sludge is low and the time required to achieve stable and complete nitrification is generally long. Continuous stirred tank reactor (CSTR), another commonly used floccular sludge reactor configuration, is operated with continuous feeding and draining on top of complete mixing of the reacting mixture and is suitable for wastewater treatment with stable quality and large quantity, with advantages of simple operation and easy control (Ji et al. 2019). A modeling study by Zhu et al. (2023) found that CSTR could ensure low ammonium levels in the reactor by continuous slow feeding, which was more conducive to the efficient enrichment of comammox bacteria than SBR. Nevertheless, there exists a scarcity of direct experimental studies on not only the interactions between AOB and comammox bacteria but also the comparative enrichment of comammox bacteria in SBR and CSTR.

Therefore, in this study, a reactor was established and operated for 216 d in two phases, i.e., phase I as an SBR and phase II as a CSTR, to compare the enrichment efficiency of comammox bacteria. 16S rRNA gene amplicon sequencing and real-time quantitative polymerase chain reaction (qPCR) were utilized to track the succession of microbial communities in the floccular sludge during the two phases, while metagenomic sequencing was employed to validate the presence and taxonomy of the enriched comammox bacteria. Moreover, batch tests were conducted using the floccular sludge enriched in this study and the *Nitrosomonas*-dominant floccular sludge enriched by Chen et al. (2024), aiming to investigate the competitive relationship between comammox bacteria and AOB for substrates under different levels of substrates. The results would provide further insights into the efficient obtainment of comammox bacteria-dominant floccular sludge and the interactions between AOB and comammox bacteria in the floccular sludge, thus contributing to the future development of comammox-inclusive BNR technologies for sustainable wastewater treatment.

## 2 Results and discussion

### 2.1 Reactor performance and microbial community succession

**Fig. 1:**
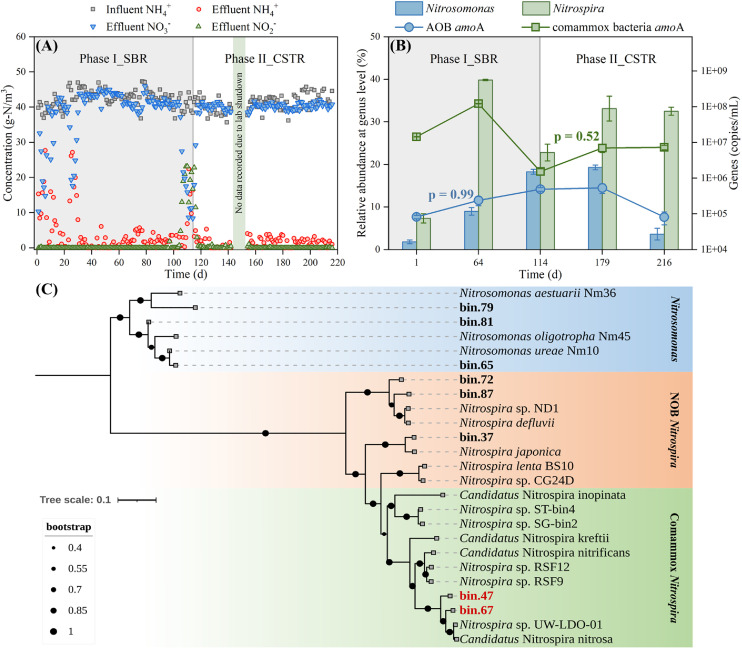
(A) Influent and effluent compositions of reactor, (B) relative abundances of *Nitrosomonas* and *Nitrospira* and numbers of *amo*A functional genes in floccular sludge, and (C) phylogenetic tree of nitrifying microorganism bins in this study and representative *Nitrosomonas* and *Nitrospira* collected from NCBI database. P in (B) represents Pearson's partial correlation coefficient between the relative abundance of each identified nitrifier at the genus level and the number of associated *amo*A functional genes.

Fig. 1A and B describes the changes in the influent/effluent compositions and the microbial community succession in the floccular sludge, respectively, with the reactor operated as an SBR during phase I (Day 1-Day 114) and a CSTR during phase II (Day 114-Day 216).

During the first 30 d of phase I, the influent ammonium was not fully utilized following the inoculation of comammox bacteria-containing floccular sludge, probably due to the storage at −4 °C suppressing the activity of the floccular sludge. Given its favorable competitive position in the inoculated sludge, the proportion of *Nitrospira* rose from 7.3 ± 1.1 % on Day 1 to 39.8 ± 0.2 % on Day 64 and the abundance of comammox bacteria *amo*A gene increased from 1.4 ± 0.1 × 10^7^ copies/mL to 1.2 ± 0.1 × 10^8^ copies/mL. It was clear that after 64-d enrichment, nitrifiers (i.e., *Nitrosomonas* + *Nitrospira*) became the dominant microorganisms in the reactor (see Fig. S1 for more details). At this point, the influent ammonium was mostly oxidized to nitrate, forming a stable nitrification process. The barely detectable nitrite accumulation during the reactor operation might suggest a portion of NOB in the floccular sludge competing with comammox bacteria for nitrite. However, despite the continuous drop in mixed liquor suspended solids (MLSS) and mixed liquor volatile suspended solids (MLVSS) (see Fig. S2), the reactor with relatively sufficient substrates also promoted the proliferation of AOB, with the proportion of *Nitrosomonas* and the abundance of AOB *amo*A gene copies growing continuously from 1.8 ± 0.5 % and 8.3 ± 0.9 × 10^4^ copies/mL on Day 1 to 18.3 ± 0.6 % and 4.9 ± 0.5 × 10^5^ copies/mL on Day 114, respectively. The strong competition of AOB for ammonium led to a rapid decline in not only *Nitrospira*, being 22.8 ± 2.0 %, but also comammox bacteria, whose *amo*A gene level dropped dramatically to 1.5 ± 0.3 × 10^6^ copies/mL on Day 114, only 1.3 % of that on Day 64. These shifts in the microbial community substantially affected the performance of the reactor. The rise in the relative abundance of *Nitrosomonas* was coupled with a gradual increase in nitrite accumulation and a decrease in nitrate production, resulting in a full nitrification efficiency of 8.4–17.5 % between Day 109 and Day 114.

Following the transition to the CSTR mode in phase II from Day 114, the incomplete utilization of the influent ammonium quickly disappeared. Negligible nitrite accumulation was noted in the reactor and the effluent ammonium was mostly below 4.0 g-N/m^3^, corresponding to a full nitrification efficiency of ∼95.0 %. *Nitrospira*/comammox bacteria gained the competitive advantage, with the proportion of *Nitrospira* and the abundance of comammox bacteria *amo*A gene copies first increasing to 33.1 ± 2.9 % and 7.0 ± 1.8 × 10^6^ copies/mL, respectively, on Day 179. Although the abundance of *Nitrospira* remained relatively stable on Day 216, there was a slight increase in comammox bacteria *amo*A gene copies, which might suggest a rise in the relative abundance of comammox *Nitrospira* and a slight drop in the relative abundance of NOB *Nitrospira*. Comparatively, the proportion of *Nitrosomonas* and the abundance of AOB *amo*A gene copies first remained stable till Day 179 and decreased sharply to 3.3 ± 1.9 % and 8.1 ± 3.2 × 10^4^ copies/mL on Day 216, respectively. The sharp decrease in *Nitrosomonas* and AOB *amo*A gene copies might be due to the insufficient ammonium as a common substrate resulting from the increased presence of comammox bacteria. Meanwhile, both MLSS and MLVSS picked up gradually (see Fig. S2).

We employed Pearson's partial correlation analysis to investigate the strength of association between proportions of nitrifiers at the genus level and abundances of *amo*A functional genes. As shown in Fig. 1B, a robust association was observed between the proportion of *Nitrosomonas* and the abundance of AOB *amo*A gene copies (p = 0.99). However, the correlation between the proportion of *Nitrospira* and the abundance of comammox bacteria *amo*A gene copies was moderate (p = 0.52), indicating the presence of a portion of NOB in the enriched floccular sludge that constituted the measured genus *Nitrospira*.

Metagenomic sequencing was then conducted on the biomass samples collected in phase II on Day 216 to further validate the presence and taxonomy of comammox bacteria on top of the coexisting AOB and NOB in the final enriched floccular sludge. Metagenomic binning recovered two bins belonging to comammox *Nitrospira*, named bin.47 and bin.67, respectively. As indicated by the phylogenetic tree in Fig. 1C, both bin.47 and bin.67 were closely placed near *Nitrospira nitrosa*, belonging to *Nitrospira* clade A. This finding was consistent with previous reports (Camejo et al. 2017; Xu et al. 2022; Zheng et al. 2023) that the majority of comammox bacteria found in wastewater treatment systems belong to the *Nitrospira* clade A and are closely associated with *Nitrospira nitrosa*. Bin.37 and bin.72/87 were found to be closely related to *Nitrospira japonica* and *Nitrospira defluvii*, respectively, both of which are strict NOB and have been reported in activated sludge at WWTPs (Nowka et al. 2015; Spieck et al. 2006; Ushiki et al. 2013). Three other bins (i.e., bin.65, bin.79, and bin.81) were identified within *Nitrosomonas*: bin.65 was closely associated with *Nitrosomonas ureae*, while bin.79 and bin.81 exhibited a close association with *Nitrosomonas aestuarii* and *Nitrosomonas oligotropha*, respectively. The presence of NOB might mainly grow on nitrite produced by AOB or/and comammox bacteria. Even so, it is evident in Fig. 1B that compared with SBR, CSTR was more suitable for the growth of comammox bacteria and tended to wash out AOB, which was consistent with Zhu et al. (2023) who reported through model-based simulations that CSTR was a better choice to enrich comammox bacteria in the floccular sludge than SBR. Therefore, if the operation of the reactor as a CSTR were to continue, we could expect the decreasing presence of AOB accompanied by an elevating abundance of comammox bacteria in the floccular sludge.

### 2.2 Ammonium oxidation features of enriched floccular sludge

Dedicated batch tests at different initial ammonium and DO levels were performed using the floccular sludge enriched in CSTR with significantly more comammox bacteria than AOB on Day 216. To verify the competitive relationship between AOB and comammox bacteria in low-substrate environments, batch tests with *Nitrosomonas*-dominant sludge taken from the lab-scale 8 L SBR reported by Chen et al. (2024) which was inoculated with the same full-scale activated sludge as Hou et al. (2024) and fed with synthetic wastewater containing ∼400.0 g-N/m^3^ NH_4_^+^ were also conducted. It should be noted that *Nitrospira* and *Nitrosomonas* were the most abundant microorganisms in the two types of floccular sludge used to perform the batch tests, respectively, and that the overall relative abundance of nitrifiers (i.e., *Nitrosomonas* + *Nitrospira*) in *Nitrosomonas*-dominant sludge (i.e., 36.1 % ± 2.3 %) was comparable to that in the floccular sludge enriched in this study (i.e., 35.5 % ± 0.2 %) (see Fig. S3).

**Fig. 2:**
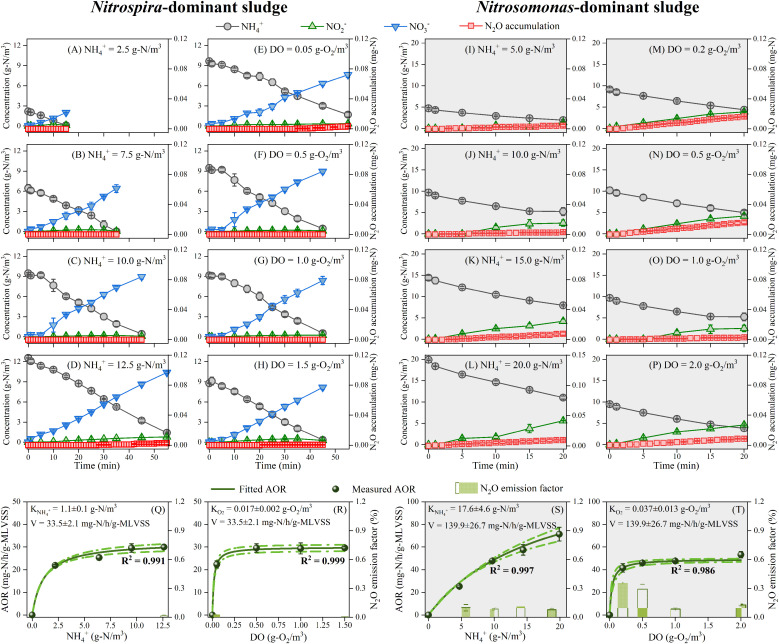
Profiles of ammonium, nitrite, and nitrate concentrations, and N_2_O accumulation during batch tests with (A-D, I-L) different initial ammonium concentrations at DO concentration of 0.5 g-O_2_/m^3^ and (E-H, M-P) different DO concentrations at initial ammonium concentration of 10.0 g-N/m^3^. Batch tests were conducted using *Nitrospira*-dominant sludge (left panel) and *Nitrosomonas-*dominant sludge (right panel). Maximum AORs and N_2_O emission factors of (Q-R) *Nitrospira*-dominant sludge and (S-T) *Nitrosomonas*-dominant sludge at different levels of ammonium and DO. The green solid and dotted lines in Q-T indicate the best fit of data to the Michaelis-Menten equation (with R^2^ showing the goodness of fit) based on nonlinear regression and the resultant 95 % confidence bands, respectively.

Clear disparities were observed between batch tests with different initial ammonium and DO levels based on *Nitrospira*-dominant sludge and *Nitrosomonas*-dominant sludge (see Fig. 2A–P). Ammonium was mainly converted to nitrate by *Nitrospira*-dominant sludge with minimal nitrite production and N_2_O accumulation, while *Nitrosomonas*-dominant sludge converted most ammonium to nitrite, accompanied by varying but significant N_2_O accumulation.

The calculated maximum AORs and N_2_O emission factors are compiled in Fig. 2Q–T. Overall, AORs increased with the initial ammonium and DO concentrations and stabilized after reaching certain values, except for the ammonium batch tests using *Nitrosomonas*-dominant sludge. Based on the calculated maximum AORs and the commonly-applied Michaelis-Menten equation (Nie et al. 2023; Wu et al. 2024), the apparent ammonium affinity constant (K_NH4__+_), apparent oxygen affinity constant (K_O2_), and maximum AOR without substrate limitations (V), were fitted to be 1.1 ± 0.1 g-N/m^3^, 0.017 ± 0.002 g-O_2_/m^3^, and 33.5 ± 2.1 mg-N/h/g-MLVSS, respectively, for *Nitrospira*-dominant sludge, and 17.6 ± 4.6 g-N/m^3^, 0.037 ± 0.013 g-O_2_/m^3^, and 139.9 ± 26.7 mg-N/h/g-MLVSS, respectively, for *Nitrosomonas*-dominant sludge. The values of kinetic parameters fitted to *Nitrospira*-dominant sludge and *Nitrosomonas*-dominant sludge were significantly different. Compared with *Nitrosomonas*-dominant sludge, *Nitrospira*-dominated sludge exhibited a lower ammonium oxidation rate but a significantly higher affinity for ammonium and DO, which may provide a plausible explanation for the fact that *Nitrosomonas* was outcompeted by *Nitrospira*/comammox bacteria and effectively washed out in CSTR.

Compared with *Nitrosomonas*-dominant sludge, *Nitrospira*-dominant sludge produced/emitted much less N_2_O (i.e., 0.08–0.35 % vs. up to 0.03 %). This phenomenon was consistent with the reported absence of NOR in comammox bacteria to generate N_2_O directly through biological processes (Kits et al. 2019; Zhu et al. 2024).

### 2.3 Key implications of this study

Based on the differences in kinetics, nitrifiers can be classified into r-strategists (i.e., with a high maximum growth rate but high substrate affinity constants) and K-strategists (i.e., with a low maximum growth rate but low substrate affinity constants) (Schramm et al. 1999; Yu et al. 2020). In this study, K_NH4__+_, K_O2_, and V of *Nitrospira*-dominant sludge obtained were lower than those counterparts of *Nitrosomonas*-dominant sludge (i.e., 1.1 ± 0.1 g-N/m^3^ vs. 17.6 ± 4.6 g-N/m^3^, 0.017 ± 0.002 g-O_2_/m^3^ vs. 0.037 ± 0.013 g-O_2_/m^3^, and 33.5 ± 2.1 mg-N/h/g-MLVSS vs. 139.9 ± 26.7 mg-N/h/g-MLVSS, respectively), in line with the fact that *Nitrospira* belongs to K-strategists (Poghosyan et al. 2020; Shao and Wu 2021) while *Nitrosomonas* is typically a r-strategist (Liu and Wang 2013; Yu et al. 2020). Wu et al. (2019) noted that when resources were sufficient, fast-growing r-strategists tended to outcompete K-strategists. Notably, the difference in K_O2_ between *Nitrospira*-dominant sludge and *Nitrosomonas*-dominant sludge obtained in this study was relatively minor, while the difference in K_NH__4__+_ between the two was rather substantial. This suggested that ammonium might play a more profound role in regulating the competition between comammox bacteria and AOB than DO. Therefore, the ammonium concentration in the reactor could be the key factor driving the selective enrichment of comammox bacteria in the floccular sludge.

**Fig. 3:**
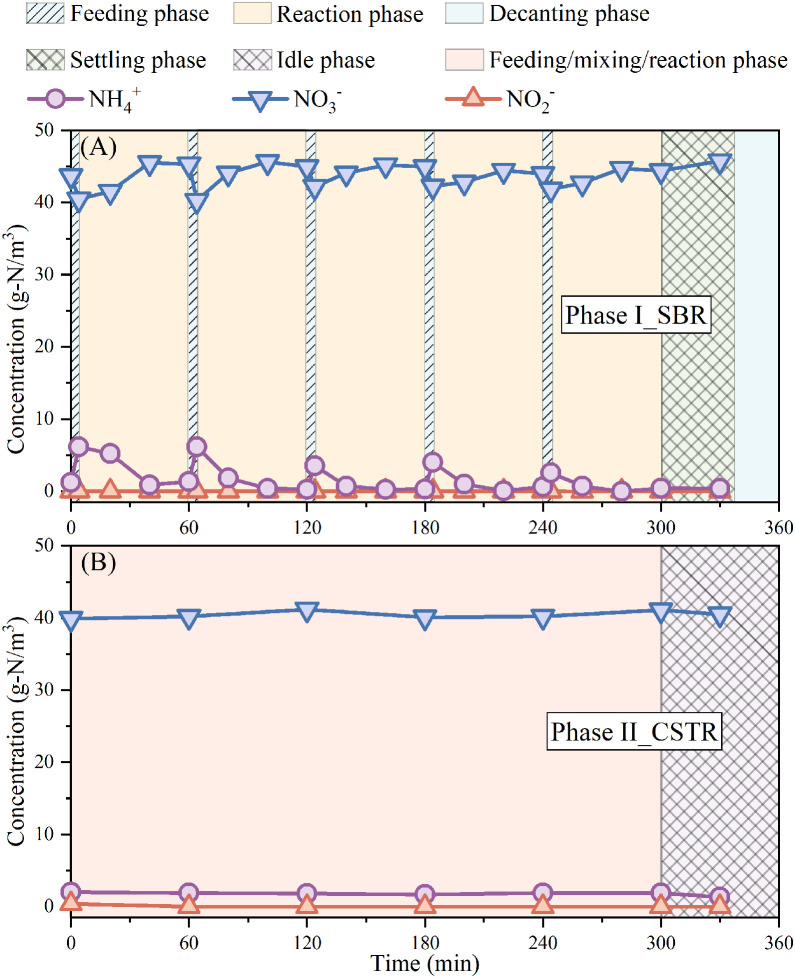
Typical cycle profiles of ammonium, nitrite, and nitrate when reactor was operated as (A) an SBR in phase I and (B) a CSTR in phase II.

In fact, a substantial difference was observed in the *in-situ* ammonium level between SBR and CSTR. It can be seen in Fig. 3 that ammonium concentration fluctuated between 1.0 and 6.0 g-N/m^3^ in SBR, while it was constantly < 2.0 g-N/mm^3^ in CSTR. Under such low ammonium conditions in CSTR, comammox bacteria with a highly oligotrophic lifestyle and a high affinity for substrates (Koch et al. 2018; Sakoula et al. 2020) tended to exhibit stronger competitiveness, thus providing a more challenging environment for AOB (i.e., *Nitrosomonas*) as an r-strategist. This is consistent with Yu et al. (2020) who pointed out that SBR was more conducive to the proliferation of r-AOB (i.e., *Nitrosomonas*) and Winkler et al. (2015) who claimed that K-strategists were more suited for growth in CSTR. It should be highlighted that the coexisting *Candidatus Nitrospira defluvii* has also been reported to be a K-strategist (Liu et al. 2019; Park et al. 2017; Wegen et al. 2019). This might account for the retention of NOB in the enriched floccular sludge despite the general trace presence of nitrite during the operation of the reactor (see Fig. 1A). However, it has been demonstrated that both comammox bacteria and *Candidatus Nitrospira defluvii* that survived in CSTR could oxidize nitrite to nitrate using oxygen (Koch et al. 2018; Park et al. 2017). The possible competitive relationship between comammox bacteria and *Candidatus Nitrospira defluvii* for nitrite suggests the significance of the subsequent work to reveal the interaction between comammox bacteria and NOB. Overall, different from the modeling approach adopted by Zhu et al. (2023), this study compared the efficiency of SBR and CSTR in enriching comammox bacteria directly through experimental demonstrations and proved that CSTR could enrich comammox bacteria while washing AOB out of the floccular sludge. However, this finding could be further verified through species-level analyses in subsequent work.

Since SBR was clearly suited to enrich *Nitrosomonas*-dominant sludge, it should provide an ideal choice of reactor configurations to achieve stable partial nitrification (PN), which could be coupled with anammox to form an autotrophic BNR technology for the treatment of wastewater with a low carbon to nitrogen ratio (Zhang et al. 2017), i.e. PN/A, characterized by low oxygen requirement and no external carbon demand (Chen et al. 2020). Comparatively, since CSTR was conducive to the formation of the comammox bacteria-dominated nitrification process, its coupling with heterotrophic denitrification or anammox might facilitate the construction of comammox-inclusive BNR technologies with low N_2_O production (Li et al. 2022; Zhu et al. 2024). Overall, this study provided further insights into the development of BNR technologies for sustainable wastewater treatment.

Still, we acknowledge that this study primarily compared the influence of different reactor operation modes on the enrichment of comammox bacteria in floccular sludge under ideal operational conditions. For instance, the influent ammonium concentration was assumed constant for the reactor, which is not the case at WWTPs. Moreover, floccular sludge-based bioreactors at some WWTPs are operated in the plug-flow mode, leading to concentration gradients in the bioreactors. Despite all, this study clearly pointed out that under the ammonium condition of mainstream wastewater (i.e., 40.0 g-N/m^3^), SBR with initially high but gradually decreasing ammonium concentrations was more suitable for the growth of AOB while CSTR with continuously low *in*-*situ* levels might be prone to the proliferation of comammox bacteria. In a word, differences in ammonium concentrations in bioreactors due to different modes of operation create different survival environments for comammox bacteria and explain the discrepancies reported in the literature concerning the presence, prevalence, and activity of comammox bacteria at WWTPs (Zheng et al. 2023; Zheng et al. 2019).

## 3 Conclusions

By operating a lab-scale reactor in two phases, this study compared the efficiency of enriching comammox bacteria in SBR and CSTR under the ammonium condition of mainstream wastewater (i.e., 40.0 g-N/m^3^). The results suggested that SBR with a fluctuating but generally higher *in-situ* ammonium concentration (i.e., 1.0–6.0 g-N/m^3^) provided a more favorable environment for the proliferation of AOB, whereas comammox bacteria with a highly oligotrophic lifestyle were more suited for growth in CSTR with a relatively lower *in-situ* ammonium level (i.e., < 2.0 g-N/m^3^). Moreover, K_NH4__+_, K_O2_, and V of *Nitrospira*-dominant sludge obtained in CSTR were lower than those of *Nitrosomonas*-dominant sludge (i.e., 1.1 ± 0.1 g-N/m^3^ vs. 17.6 ± 4.6 g-N/m^3^, 0.017 ± 0.002 g-O_2_/m^3^ vs. 0.037 ± 0.013 g-O_2_/m^3^, and 33.5 ± 2.1 mg-N/h/g-MLVSS vs. 139.9 ± 26.7 mg-N/h/g-MLVSS, respectively), proving the nature of comammox bacteria as a K-strategist.

## 4 Methods

### 4.1 Reactor operation

In this study, the comammox bacteria-containing floccular sludge previously obtained by Hou et al. (2024) that had been stored at −4 °C for two months was seeded to an 8-L reactor, resulting in an initial MLSS level of approximately 1000 g/m^3^. The operation of the reactor was divided into the following two phases:

• Phase I (Day 1-Day 114): The reactor was operated as an SBR with a 6-hour working cycle that included 300-minute feeding/reaction, 40-minute settling, and 20-minute decanting. The feeding/reaction phase with mixing was composed of 5 sequential repetitions, each consisting of 4-minute feeding followed by 56-minute reaction. Throughout the 20-minute feeding/decanting, 4 L of synthetic wastewater or supernatant was circulated in and out of the reactor, giving rise to a hydraulic retention time (HRT) of 12 h.
• Phase II (Day 114-Day 216): The reactor was operated as a CSTR by adding a membrane module for effluent draining. During the feeding/mixing/reaction, the synthetic wastewater was continuously circulated in and out of the reactor to achieve an HRT of 12 h. To avoid potential risks caused by the continuous operation, 1-hour downtime (idle phase) was also applied to the mixer, analogous to the setup for the aforementioned 6-hour working cycle of the SBR operation.

The reactor was managed by a programmable logic controller (PLC) system and placed in a laboratory at 25 ± 2 °C. Either 0.1 M HCl or NaOH was added to maintain the pH level in the reactor at 7.3 ± 0.2. The DO level was kept at 0.5 ± 0.1 g-O_2_/m^3^ by adjusting an air pump at a fixed flow rate of 0.3 L/min. The reactor was fed with NH_4_Cl at 40.0 g-N/m^3^ to simulate the ammonium level of mainstream wastewater. In addition, the synthetic wastewater composition (per liter) included 1 mL selenium wolfram solution (SWS) and 1 mL trace-element solution (TES) (Daims et al. 2015), and 998 mL macro-element solution (MES). The compositions of MES, TES, and SWS are detailed in Table S1. Since both Li et al. (2021) and Cotto et al. (2020) highlighted the significance of an extended SRT for preserving a high abundance of comammox bacteria in floccular sludge systems, no dedicated sludge wasting was set during both phases in this study.

Daily monitoring of nitrogenous compounds was conducted for both the influent and effluent. MLSS and MLVSS levels were measured occasionally throughout the operation. About 30 mL floccular sludge samples were collected from the reactor every 1–2 months and stored at −80 °C after centrifugation for microbial analyses.

### 4.2 Batch tests

At the end of either phase when there was a significantly higher presence of comammox bacteria than AOB, the floccular sludge was sampled to conduct batch tests to investigate the competitive relationship between comammox bacteria and AOB for substrates at different levels (i.e., DO of 0.05–2.0 g-O_2_/m^3^ and initial ammonium of 2.5–20.0 g-N/m^3^). The results were then compared with another set of batch tests performed with *Nitrosomonas*-dominant sludge enriched by Chen et al. (2024). Each batch test was conducted in at least duplicate in a small reactor with a working volume of 1.0 L at 25 ± 2 °C. A separate PLC system was utilized to adjust the DO level in the small reactor to the specified level while maintaining the pH level in a range of 7.5 ± 0.2 by adding 0.1 M HCl or NaOH. The mixed liquor was continuously mixed at 300 rpm using a magnetic stirrer during each batch test. Mixed liquor samples were regularly collected and filtered through 0.22 μm filters to analyze nitrogenous compounds, while N_2_O emissions in off-gas were also quantified. The designed MLVSS level was verified after each batch test.

### 4.3 Analytical methods

#### 4.3.1 Chemical analyses

Following established protocols (APHA 2007), NH_4_^+^, NO_2_^-^, NO_3_^-^, MLSS, and MLVSS concentrations were determined. The DO and pH levels were tracked using a real-time DO probe/meter (MIK-DY2016, Meacon, China) and a pH probe/meter (MIK-pH 6.0, Meacon, China), respectively. The N_2_O concentration in off-gas was continuously assessed with an infrared photometer (AO2020, ABB, Germany) and used in conjunction with the recorded off-gas flow rate to determine the total N_2_O emissions during each batch test.

Given that there was no NO_3_^-^ in the influent, the full nitrification (NH_4_^+^ → NO_3_^-^) efficiency was defined as the ratio (%) of NO_3_^-^ in the effluent to NH_4_^+^ in the influent. The maximum ammonium oxidation rate (AOR, mg-N/h/g-MLVSS) of each batch test was determined based on the nearly linear trends of decreasing NH_4_^+^ concentration versus time with the fitted rate normalized to the measured MLVSS level. The N_2_O emission factors were calculated as the total N_2_O accumulation per NH_4_^+^ removed (ΔN_2_O-N/ΔNH_4_^+^-N, %).

#### 4.3.2 Microbial analyses

##### 4.3.2.1 DNA extraction

Microbial analyses were performed on a total of 10 stored biomass samples collected during the long-term operation of the reactor. Firstly, DNA was extracted from these biomass samples using the FastDNA® Spin Kit for Soil (MP Biomedicals, USA) per the manufacturer's protocol. DNA quality and concentration were examined using 1.0 % agarose gel electrophoresis and the final DNA concentration was quantified on a NanoDrop® ND-2000 spectrophotometer (Thermo Scientific Inc., USA). The extracted DNA was used for subsequent 16S rRNA gene amplicon sequencing, quantification of *amo*A genes by qPCR, and metagenomic sequencing.

##### 4.3.2.2 16S rRNA gene amplicon sequencing

The bacterial 16S rRNA gene sequences (V3-V4 regions) of the extracted DNA were amplified via the primers 338F (5′-ACTCCTRCGGGAGGCAGCAG-3′) and 806R (5′-GGACTACCVGGGTATCTAAT-3′) by T100 Thermal Cycler PCR thermocycler (BIO-RAD, USA). The PCR reaction mixture consisted of 4 μL of 5 × Fast Pfu buffer, 2 μL of dNTPs (2.5 mM), 0.8 μL of forward primer (5 μM), 0.8 μL of reverse primer (5 μM), 0.4 μL of Fast Pfu polymerase, 0.2 μL of BSA, 10 ng of template DNA, and double distilled water (ddH_2_O) in a final volume of 20 µL. The PCR amplification cycle conditions are as follows: initial denaturation at 95 °C for 3 min, followed by 27 cycles of denaturation at 95 °C for 30 s, annealing at 55 °C for 30 s, and extension at 72 °C for 45 s, with a single final extension at 72 °C for 10 min, and ending at 10 °C. Amplification was checked for all biomass samples in triplicate. The PCR products were extracted from a 2 % agarose gel, purified using the PCR Clean-Up Kit (YuHua, China) according to the manufacturer's instructions, and quantified with Qubit 4.0 (Thermo Fisher Scientific, USA). Purified amplicons were pooled in equimolar amounts and paired-end sequenced using the Illumina Nextseq2000 platform (Illumina, USA) according to the manufacturer's protocols by Majorbio Bio-Pharm Technology Co. Ltd. (China).

##### 4.3.2.3 Quantification of *amo*A genes by qPCR

qPCR was performed by ChamQ SYBR Color qPCR Master Mix (2X) quantitative PCR reagent (Vazyme, China) on ABI7300 fluorescence quantitative PCR instrument (Applied Biosystems, USA). The used qPCR primers included amoA-1F/amoA-2R for *amo*A genes of AOB (Rotthauwe et al. 1997) and comamoA AF/comamoA SR for *amo*A genes of comammox bacteria (Wang et al. 2018). A pre-experiment on each microbial DNA was performed before qPCR to determine the purity of each gene for successful quantitative amplification.

##### 4.3.2.4 Metagenomic sequencing and phylogenetic analysis

DNA extract was fragmented into fragments with an average size of ∼350 bp using Covaris M220 (Gene Co., Ltd., China) and used to construct paired-end libraries using NEXTFLEX® Rapid DNA-Seq (Bioo Scientific, USA). Adapters containing a full set of sequencing primer hybridization sites were ligated to the blunt ends of the fragments. Paired-end sequencing was performed at Majorbio Bio-Pharm Technology Co., Ltd. (China) using the NovaSeq 6000 S4 Reagent Kit v1.5 (300 cycles) on the Illumina NovaSeq (Illumina Inc., USA) following the manufacturer's instructions.

The raw metagenomic data obtained was quality-checked using FastQC (v 0.11.7). Trimmomatic (version 0.39) was used to clean and control the raw data with parameters set to SLIDINGWINDOW:5:20 MINLEN:50 LEADING:3 TRAILING:3 (Bolger et al. 2014). Clean metagenomic reads were used to generate a set of contigs using metaSPAdes assembler (version 3.13.0) (Nurk et al. 2017). Contig binning was performed with metaWRAP (version 1.3.2) (Uritskiy et al. 2018) using the binning module (–metabat2 –maxbin2 –concoct) with default settings. MAG quality was evaluated using CheckM (version 1.2.0) (Parks et al. 2015). All MAGs were dereplicated using dRep (version 3.4.2) (Olm et al. 2017). CoverM (version 0.6.1) was used in genome mode to obtain MAG coverage. Forty-seven nonredundant MAGs were taxonomically assigned using GTDB-TK (version 2.1.1) (Chaumeil et al. 2020) based on the Genome Taxonomy Database (GTDB, release 214) (Parks et al. 2022). Eight MAGs classified to *Nitrospira* (5 MAGs) and *Nitrosomonas* (3 MAGs) were further analyzed.

To identify the sublineage-level phylogeny of *Nitrospira* and *Nitrosomonas* bins, 14 *Nitrospira* bins and 3 *Nitrosomonas* bins were downloaded from the NCBI database as references. The phylogenomic tree was constructed using GTDB-TK (version 2.1.1) (Chaumeil et al. 2020) with de_novo_wf command.

#### 4.3.3 Statistical analyses

The averages and standard deviations of nitrogen concentrations, maximum AORs and N_2_O emission factors of each batch test, *Nitrospira*/*Nitrosomonas* abundances, comammox bacteria/AOB *amo*A gene copies, and the Pearson's partial correlation coefficients (p) were directly calculated using Microsoft Excel (v.2022).

## CRediT authorship contribution statement

**Ying Zhu:** Writing – original draft, Visualization, Software, Methodology, Investigation, Formal analysis, Data curation. **Jiaying Hou:** Writing – original draft, Visualization, Software, Methodology, Investigation, Formal analysis, Data curation. **Fangang Meng:** Investigation. **Meiying Xu:** Investigation. **Limin Lin:** Software, Formal analysis. **Linyan Yang:** Investigation. **Xueming Chen:** Writing – review & editing, Supervision, Resources, Project administration, Funding acquisition, Formal analysis, Conceptualization.

## Declaration of competing interest

The authors declare that they have no known competing financial interests or personal relationships that could have appeared to influence the work reported in this paper.

## Data Availability

Data will be made available on request

## Appendix Supplementary materials

Image, application 1

## References

[bib0001] APHA A. (2007).

[bib0002] Askari S.S., Giri B.S., Basheer F., Izhar T., Ahmad S.A., Mumtaz N. (2024). Enhancing sequencing batch reactors for efficient wastewater treatment across diverse applications: a comprehensive review. Environ. Res..

[bib0003] Bolger A.M., Lohse M., Usadel B. (2014). Trimmomatic: a flexible trimmer for Illumina sequence data. Bioinformatics.

[bib0004] Camejo P.Y., Santo Domingo J., Mcmahon K.D., Noguera D.R., Summers Z.M.J.m. (2017). Genome-enabled insights into the ecophysiology of the comammox bacterium "candidatus nitrospira nitrosa". mSystems.

[bib0005] Chaumeil P.A., Mussig A.J., Hugenholtz P., Parks D.H. (2020). GTDB-Tk: a toolkit to classify genomes with the Genome Taxonomy Database. Bioinformatics.

[bib0006] Chen B., Li F., Lin Y., Yang L., Wei W., Ni B.-J., Chen X. (2024). Degradation of chloroquine by ammonia-oxidizing bacteria: performance, mechanisms, and associated impact on N2O production. Environ. Sci. Technol..

[bib0007] Chen H., Liu T., Li J., Mao L., Ye J., Han X., Jetten M.S.M., Guo J. (2020). Larger anammox granules not only harbor higher species diversity but also support more functional diversity. Environ. Sci. Technol..

[bib0008] Cotto I., Dai Z., Huo L., Anderson C.L., Vilardi K.J., Ijaz U., Khunjar W., Wilson C., De Clippeleir H., Gilmore K., Bailey E., Pinto A.J. (2020). Long solids retention times and attached growth phase favor prevalence of comammox bacteria in nitrogen removal systems. Water Res..

[bib0009] Daims H., Lebedeva E.V., Pjevac P., Han P., Herbold C., Albertsen M., Jehmlich N., Palatinszky M., Vierheilig J., Bulaev A., Kirkegaard R.H., von Bergen M., Rattei T., Bendinger B., Nielsen P.H., Wagner M. (2015). Complete nitrification by *Nitrospira* bacteria. Nature.

[bib0010] Fujitani H., Nomachi M., Takahashi Y., Hasebe Y., Eguchi M., Tsuneda S. (2020). Successful enrichment of low-abundant comammox nitrospira from nitrifying granules under ammonia-limited conditions. FEMS Microbiol. Lett..

[bib0011] Han P., Wu D., Sun D., Zhao M., Wang M., Wen T., Zhang J., Hou L., Liu M., Klümper U., Zheng Y., Dong H.-P., Liang X., Yin G. (2021). N2O and NOy production by the comammox bacterium Nitrospira inopinata in comparison with canonical ammonia oxidizers. Water Res..

[bib0012] He S.S., Li Y.W., Mu H.B., Zhao Z.R., Wang J.W., Liu S.F., Sun Z.L., Zheng M.S. (2021). Ammonium concentration determines differential growth of comammox and canonical ammonia-oxidizing prokaryotes in soil microcosms. Appl. Soil Ecol..

[bib0013] Hou J., Zhu Y., Liu J., Lin L., Zheng M., Yang L., Wei W., Ni B.-J., Chen X. (2024). Competitive enrichment of comammox Nitrospira in floccular sludge. Water Res..

[bib0014] Huang T., Xia J., Liu T., Su Z., Guan Y., Guo J., Wang C., Zheng M. (2022). Comammox *nitrospira* bacteria are dominant ammonia oxidizers in mainstream nitrification bioreactors emended with sponge carriers. Environ. Sci. Technol..

[bib0015] Ji X., Wu Z., Sung S., Lee P.-H. (2019). Metagenomics and metatranscriptomics analyses reveal oxygen detoxification and mixotrophic potentials of an enriched anammox culture in a continuous stirred-tank reactor. Water Res..

[bib0016] Kits K.D., Jung M.-Y., Vierheilig J., Pjevac P., Sedlacek C.J., Liu S., Herbold C., Stein L.Y., Richter A., Wissel H., Brüggemann N., Wagner M., Daims H. (2019). Low yield and abiotic origin of N2O formed by the complete nitrifier *Nitrospira* inopinata. Nat. Commun..

[bib0017] Kits K.D., Sedlacek C.J., Lebedeva E.V., Han P., Bulaev A., Pjevac P., Daebeler A., Romano S., Albertsen M., Stein L.Y., Daims H., Wagner M. (2017). Kinetic analysis of a complete nitrifier reveals an oligotrophic lifestyle. Nature.

[bib0018] Koch H., van Kessel M.A.H.J., Lücker S. (2018). Complete nitrification: insights into the ecophysiology of comammox *Nitrospira*. Appl. Microbiol. Biotechnol..

[bib0019] Kwon H., Kang H.-J., Park Y., Bae J. (2023). Optimization of a sequencing batch reactor with the application of the Internet of Things. Water Res..

[bib0020] Li J., Hua Z.-S., Liu T., Wang C., Li J., Bai G., Lücker S., Jetten M.S.M., Zheng M., Guo J. (2021). Selective enrichment and metagenomic analysis of three novel comammox *Nitrospira* in a urine-fed membrane bioreactor. ISME Commun..

[bib0021] Li X., Wang G., Chen J., Zhou X., Liu Y. (2022). Deciphering the concurrence of comammox, partial denitrification and anammox in a single low-oxygen mainstream nitrogen removal reactor. Chemosphere.

[bib0022] Liu G., Wang J. (2013). Long-term low DO enriches and shifts nitrifier community in activated sludge. Environ. Sci. Technol..

[bib0023] Liu W., Chen W., Yang D., Shen Y. (2019). Functional and compositional characteristics of nitrifiers reveal the failure of achieving mainstream nitritation under limited oxygen or ammonia conditions. Bioresour. Technol..

[bib0024] Lu X., D S Pereira T., Al-Hazmi H.E., Majtacz J., Zhou Q., Xie L., Makinia J (2018). Model-based evaluation of N2O production pathways in the Anammox-enriched granular sludge cultivated in a sequencing batch reactor. Environ. Sci. Technol..

[bib0025] Nie W.-B., Xie G.-J., Tan X., Ding J., Lu Y., Chen Y., Yang C., He Q., Liu B.-F., Xing D., Ren N. (2023). Microbial niche differentiation during nitrite-dependent Anaerobic methane oxidation. Environ. Sci. Technol..

[bib0026] Nowka B., Off S., Daims H., Spieck E. (2015). Improved isolation strategies allowed the phenotypic differentiation of two Nitrospira strains from widespread phylogenetic lineages. FEMS Microbiol. Ecol..

[bib0027] Nurk S., Meleshko D., Korobeynikov A., Pevzner P.A. (2017). metaSPAdes: a new versatile metagenomic assembler. Genome Res..

[bib0028] Olm M.R., Brown C.T., Brooks B., Banfield J.F. (2017). dRep: a tool for fast and accurate genomic comparisons that enables improved genome recovery from metagenomes through de-replication. ISME J..

[bib0029] Palomo A., Dechesne A., Pedersen A.G., Smets B.F. (2022). Genomic profiling of *Nitrospira* species reveals ecological success of comammox Nitrospira. Microbiome.

[bib0030] Park M.-R., Park H., Chandran K. (2017). Molecular and kinetic characterization of planktonic *nitrospira* spp. Selectively enriched from activated sludge. Environ. Sci. Technol..

[bib0031] Parks D.H., Chuvochina M., Rinke C., Mussig A.J., Chaumeil P.A., Hugenholtz P. (2022). GTDB: an ongoing census of bacterial and archaeal diversity through a phylogenetically consistent, rank normalized and complete genome-based taxonomy. Nucl. Acid. Res..

[bib0032] Parks D.H., Imelfort M., Skennerton C.T., Hugenholtz P., Tyson G.W. (2015). CheckM: assessing the quality of microbial genomes recovered from isolates, single cells, and metagenomes. Genome Res..

[bib0033] Poghosyan L., Koch H., Frank J., van Kessel M.A.H.J., Cremers G., van Alen T., Jetten M.S.M., Op den Camp H.J.M., Lücker S. (2020). Metagenomic profiling of ammonia- and methane-oxidizing microorganisms in two sequential rapid sand filters. Water Res..

[bib0034] Rotthauwe J.H., Witzel K.P., Liesack W. (1997). The ammonia monooxygenase structural gene amoA as a functional marker: molecular fine-scale analysis of natural ammonia-oxidizing populations. Appl. Environ. Microbiol..

[bib0035] Sakoula D., Koch H., Frank J., Jetten M.S.M., van Kessel M.A.H.J., Lücker S. (2020). Enrichment and physiological characterization of a novel comammox *Nitrospira* indicates ammonium inhibition of complete nitrification. ISME J..

[bib0036] Sato Y., Tanaka E., Hori T., Futamata H., Murofushi K., Takagi H., Akachi T., Miwa T., Inaba T., Aoyagi T., Habe H. (2021). Efficient conversion of organic nitrogenous wastewater to nitrate solution driven by comammox *Nitrospira*. Water Res..

[bib0037] Schramm A., de Beer D., van den Heuvel Johan C., Ottengraf S., Amann R. (1999). Microscale distribution of populations and activities of *nitrosospira* and *nitrospira* spp. Along a macroscale gradient in a nitrifying bioreactor: quantification by In situ hybridization and the use of microsensors. Appl. Environ. Microbiol..

[bib0038] Shao Y.-H., Wu J.-H. (2021). Comammox *nitrospira* species dominate in an efficient partial nitrification–Anammox bioreactor for treating ammonium at low loadings. Environ. Sci. Technol..

[bib0039] Shi M., Li J., Gao R., Song X., Wang G., Gao Y., Yan S. (2023). Contrasting effects of elevated CO2 on autotrophic prokaryotes with different CO2 fixation strategies in tea plantation soil. Biol. Fertil. Soil..

[bib0040] Spasov E., Tsuji J.M., Hug L.A., Doxey A.C., Sauder L.A., Parker W.J., Neufeld J.D. (2020). High functional diversity among *Nitrospira* populations that dominate rotating biological contactor microbial communities in a municipal wastewater treatment plant. ISME J..

[bib0041] Spieck E., Hartwig C., McCormack I., Maixner F., Wagner M., Lipski A., Daims H. (2006). Selective enrichment and molecular characterization of a previously uncultured Nitrospira-like bacterium from activated sludge. Environ. Microbiol..

[bib0042] Uritskiy G.V., DiRuggiero J., Taylor J. (2018). MetaWRAP-a flexible pipeline for genome-resolved metagenomic data analysis. Microbiome.

[bib0043] Ushiki N., Fujitani H., Aoi Y., Tsuneda S. (2013). Isolation of *Nitrospira* belonging to Sublineage II from a wastewater treatment plant. Microb. Environ..

[bib0044] van Kessel M.A.H.J., Speth D.R., Albertsen M., Nielsen P.H., Op den Camp H.J.M., Kartal B., Jetten M.S.M., Lücker S. (2015). Complete nitrification by a single microorganism. Nature.

[bib0045] Vilardi K., Cotto I., Bachmann M., Parsons M., Klaus S., Wilson C., Bott C.B., Pieper K.J., Pinto A.J. (2023). Co-occurrence and cooperation between comammox and anammox bacteria in a full-scale attached growth municipal wastewater treatment process. Environ. Sci. Technol..

[bib0046] Wang M., Huang G., Zhao Z., Dang C., Liu W., Zheng M. (2018). Newly designed primer pair revealed dominant and diverse comammox amoA gene in full-scale wastewater treatment plants. Bioresour. Technol..

[bib0047] Wang Y., Ma L., Mao Y., Jiang X., Xia Y., Yu K., Li B., Zhang T. (2017). Comammox in drinking water systems. Water Res..

[bib0048] Wegen S., Nowka B., Spieck E., Liu S.-J. (2019). Low temperature and neutral pH define “*Candidatus* Nitrotoga sp.” as a competitive nitrite oxidizer in coculture with *Nitrospira defluvii*. Appl. Environ. Microbiol..

[bib0049] Winkler M.-K.H., Le Q.H., Volcke E.I.P. (2015). Influence of partial denitrification and mixotrophic growth of NOB on microbial distribution in aerobic granular sludge. Environ. Sci. Technol..

[bib0050] Wu H., Nie W.-B., Tan X., Xie G.-J., Qu H., Zhang X., Xian Z., Dai J., Yang C., Chen Y. (2024). Different oxygen affinities of methanotrophs and Comammox *Nitrospira* inform an electrically induced symbiosis for nitrogen loss. Water Res..

[bib0051] Wu L., Ning D., Zhang B., Li Y., Zhang P., Shan X., Zhang Q., Brown M.R., Li Z., Van Nostrand J.D., Ling F., Xiao N., Zhang Y., Vierheilig J., Wells G.F., Yang Y., Deng Y., Tu Q., Wang A., Acevedo D., Agullo-Barcelo M., Alvarez P.J.J., Alvarez-Cohen L., Andersen G.L., de Araujo J.C., Boehnke K.F., Bond P., Bott C.B., Bovio P., Brewster R.K., Bux F., Cabezas A., Cabrol L., Chen S., Criddle C.S., Deng Y., Etchebehere C., Ford A., Frigon D., Sanabria J., Griffin J.S., Gu A.Z., Habagil M., Hale L., Hardeman S.D., Harmon M., Horn H., Hu Z., Jauffur S., Johnson D.R., Keller J., Keucken A., Kumari S., Leal C.D., Lebrun L.A., Lee J., Lee M., Lee Z.M.P., Li Y., Li Z., Li M., Li X., Ling F., Liu Y., Luthy R.G., Mendonça-Hagler L.C., de Menezes F.G.R., Meyers A.J., Mohebbi A., Nielsen P.H., Ning D., Oehmen A., Palmer A., Parameswaran P., Park J., Patsch D., Reginatto V., de los Reyes F.L., Rittmann B.E., Noyola A., Rossetti S., Shan X., Sidhu J., Sloan W.T., Smith K., de Sousa O.V., Stahl D.A., Stephens K., Tian R., Tiedje J.M., Tooker N.B., Tu Q., Van Nostrand J.D., De los Cobos Vasconcelos D., Vierheilig J., Wagner M., Wakelin S., Wang A., Wang B., Weaver J.E., Wells G.F., West S., Wilmes P., Woo S.-G., Wu L., Wu J.-H., Wu L., Xi C., Xiao N., Xu M., Yan T., Yang Y., Yang M., Young M., Yue H., Zhang B., Zhang P., Zhang Q., Zhang Y., Zhang T., Zhang Q., Zhang W., Zhang Y., Zhou H., Zhou J., Wen X., Curtis T.P., He Q., He Z., Brown M.R., Zhang T., He Z., Keller J., Nielsen P.H., Alvarez P.J.J., Criddle C.S., Wagner M., Tiedje J.M., He Q., Curtis T.P., Stahl D.A., Alvarez-Cohen L., Rittmann B.E., Wen X., Zhou J (2019). Global diversity and biogeography of bacterial communities in wastewater treatment plants. Nat. Microbiol..

[bib0052] Xu S., Chai W., Xiao R., Smets B.F., Palomo A., Lu H. (2022). Survival strategy of comammox bacteria in a wastewater nutrient removal system with sludge fermentation liquid as additional carbon source. Sci. Tot. Environ..

[bib0053] Yu L., Chen S., Chen W., Wu J. (2020). Experimental investigation and mathematical modeling of the competition among the fast-growing “r-strategists” and the slow-growing “K-strategists” ammonium-oxidizing bacteria and nitrite-oxidizing bacteria in nitrification. Sci. Tot. Environ..

[bib0054] Yu Y., Han P., Zhou L.-J., Li Z., Wagner M., Men Y. (2018). Ammonia monooxygenase-mediated cometabolic biotransformation and hydroxylamine-mediated abiotic transformation of micropollutants in an AOB/NOB coculture. Environ. Sci. Technol..

[bib0055] Zhang X., Zhou Y., Zhang N., Zheng K., Wang L., Han G., Zhang H. (2017). Short-term and long-term effects of Zn (II) on the microbial activity and sludge property of partial nitrification process. Bioresour. Technol..

[bib0056] Zhao J., Zheng M., Su Z., Liu T., Li J., Guo J., Yuan Z., Hu S. (2022). Selective enrichment of Comammox *Nitrospira* in a moving bed biofilm reactor with sufficient oxygen supply. Environ. Sci. Technol..

[bib0057] Zheng M., Tian Z., Chai Z., Zhang A., Gu A., Mu G., Wu D., Guo J. (2023). Ubiquitous occurrence and functional dominance of comammox *Nitrospira* in full-scale wastewater treatment plants. Water Res..

[bib0058] Zheng M., Wang M., Zhao Z., Zhou N., He S., Liu S., Wang J., Wang X. (2019). Transcriptional activity and diversity of comammox bacteria as a previously overlooked ammonia oxidizing prokaryote in full-scale wastewater treatment plants. Sci. Tot. Environ..

[bib0059] Zhu G., Wang X., Wang S., Yu L., Armanbek G., Yu J., Jiang L., Yuan D., Guo Z., Zhang H., Zheng L., Schwark L., Jetten M.S.M., Yadav A.K., Zhu Y.-G. (2022). Towards a more labor-saving way in microbial ammonium oxidation: a review on complete ammonia oxidization (comammox). Sci. Tot. Environ..

[bib0060] Zhu Y., Hou J., Liu J., Huo P., Yang L., Zheng M., Wei W., Ni B.-J., Chen X. (2023). Model-based development of strategies enabling effective enrichment and application of comammox bacteria in floccular sludge under mainstream conditions. Sci. Tot. Environ..

[bib0061] Zhu Y., Hou J., Meng F., Lu H., Zhang Y., Ni B.-J., Chen X. (2024). Role of comammox bacteria in granular bioreactor for nitrogen removal via partial nitritation/anammox. Bioresour. Technol..

