# Supplementary material for: Comparative enrichment of complete ammonium oxidation bacteria in floccular sludge reactors: Sequencing batch reactor vs. continuous stirred tank reactor
Source: Water Res X. 2025 Jan 22;27:100305. doi: 10.1016/j.wroa.2025.100305 (PMC11802381; doi:10.1016/j.wroa.2025.100305)
Supplement: Supplementary file 1 [file mmc1.docx]

# Supporting Information *for*

**Comparative enrichment of complete ammonium oxidation bacteria in** **floccular sludge reactors: sequencing batch reactor vs.** **continuous stirred tank reactor**

# Ying Zhu^1^*, Jiaying Hou^1^*, Fangang Meng^2^, Meiying Xu^3^, Limin Lin^4^, Linyan Yang^5^, Xueming Chen^1,2,3#^

^1^College of Environment and Safety Engineering, Fuzhou University, Fuzhou 350116, China

^2^Guangdong Provincial Key Laboratory of Environmental Pollution Control and Remediation Technology (Sun Yat-sen University), Guangzhou 510275, China

^3^State Key Laboratory of Applied Microbiology Southern China, Institute of Microbiology, Guangdong Academy of Sciences, Guangzhou 510070, China

^4^School of Engineering, Westlake University, Hangzhou, Zhejiang 310030, China

^5^School of Resources and Environmental Engineering, East China University of Science and Technology, Shanghai 200237, China

*****These authors contributed equally to this study.

**^#^**Corresponding author:

Dr. Xueming Chen, E-mail: [xuem.chen@hotmail.com](mailto:xuem.chen@hotmail.com)

Number of pages: **4** Number of figures: **3** Number of tables: **1**

| **Table S1.** Details of MES, TES, and SWS | | |
| --- | --- | --- |
|  | **Composition** | **Concentration** |
| MES | KCl | 75.0 mg/L |
|  | KH_2_PO_4_ | 50.0 mg/L |
|  | NaCl | 584.0 mg/L |
|  | MgSO_4_·7H_2_O | 50.0 mg/L |
|  | CaCO_3_ | 170.0 mg/L |
|  | NH_4_Cl | 153.0 mg/L |
| TES | CuCl_2_·2H_2_O | 20.0 mg/L |
|  | CoCl_2_·6H_2_O | 80.0 mg/L |
|  | Na_2_MoO_4_·2H_2_O | 72.6 mg/L |
|  | ZnCl_2_ | 70.0 mg/L |
|  | FeSO_4_·7H_2_O | 1000.0 mg/L |
|  | NiCl_2_·6H_2_O | 24.0 mg/L |
|  | MnSO_4_·1H_2_O | 34.4 mg/L |
|  | H_3_BO_3_ | 50.0 mg/L |
|  | HCl (concentrated) | 2.5 mg/L |
| SWS (per liter) | NaOH | 500.0 mg/L |
|  | Na_2_SeO_3_·5H_2_O | 1.9 mg/L |
|  | Na_2_WO_4_·2H_2_O | 4.0 mg/L |

***
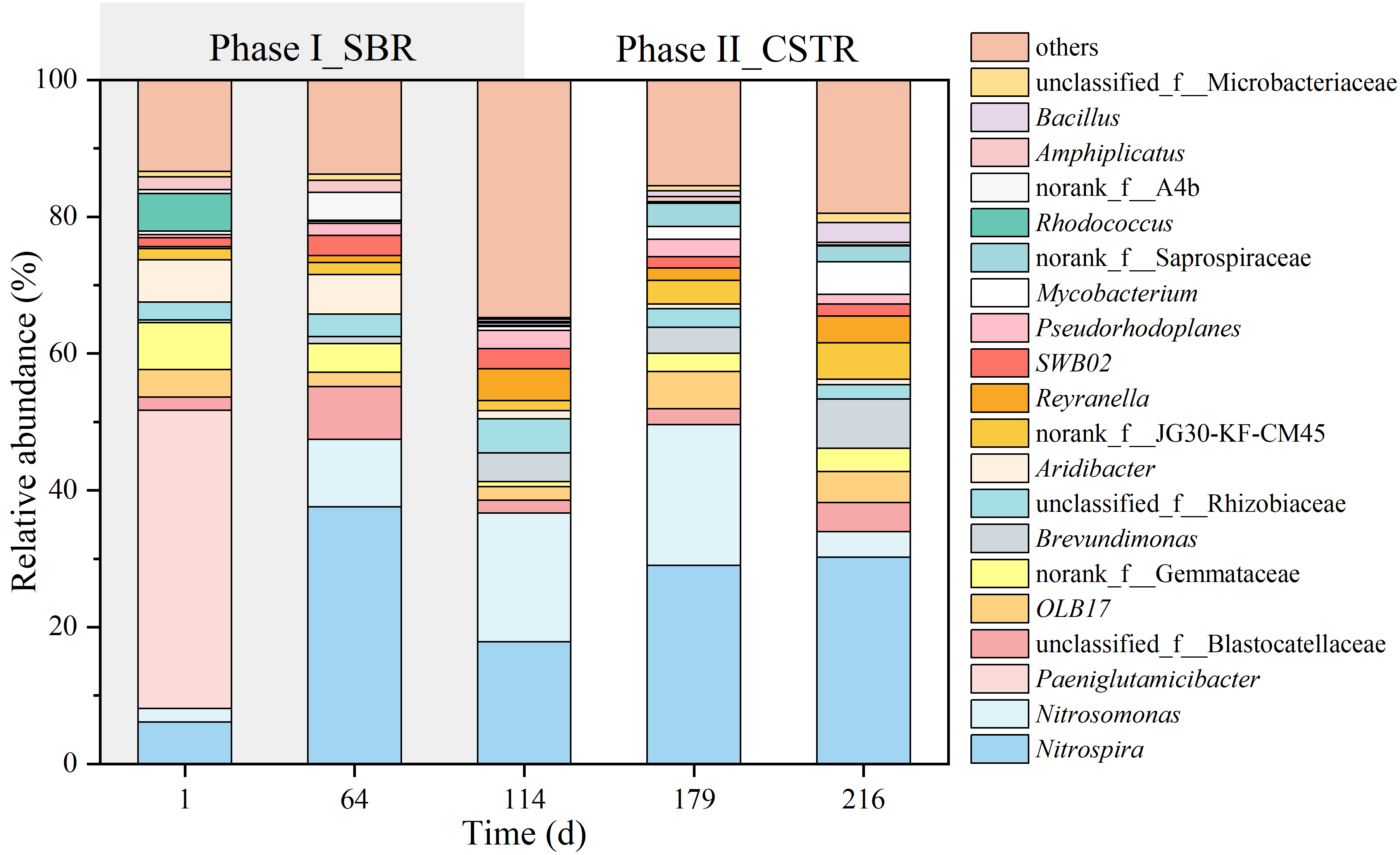
*Fig. S1.** The relative abundances of microorganisms at genus level in floccular sludge during the long-term operation of the reactor.


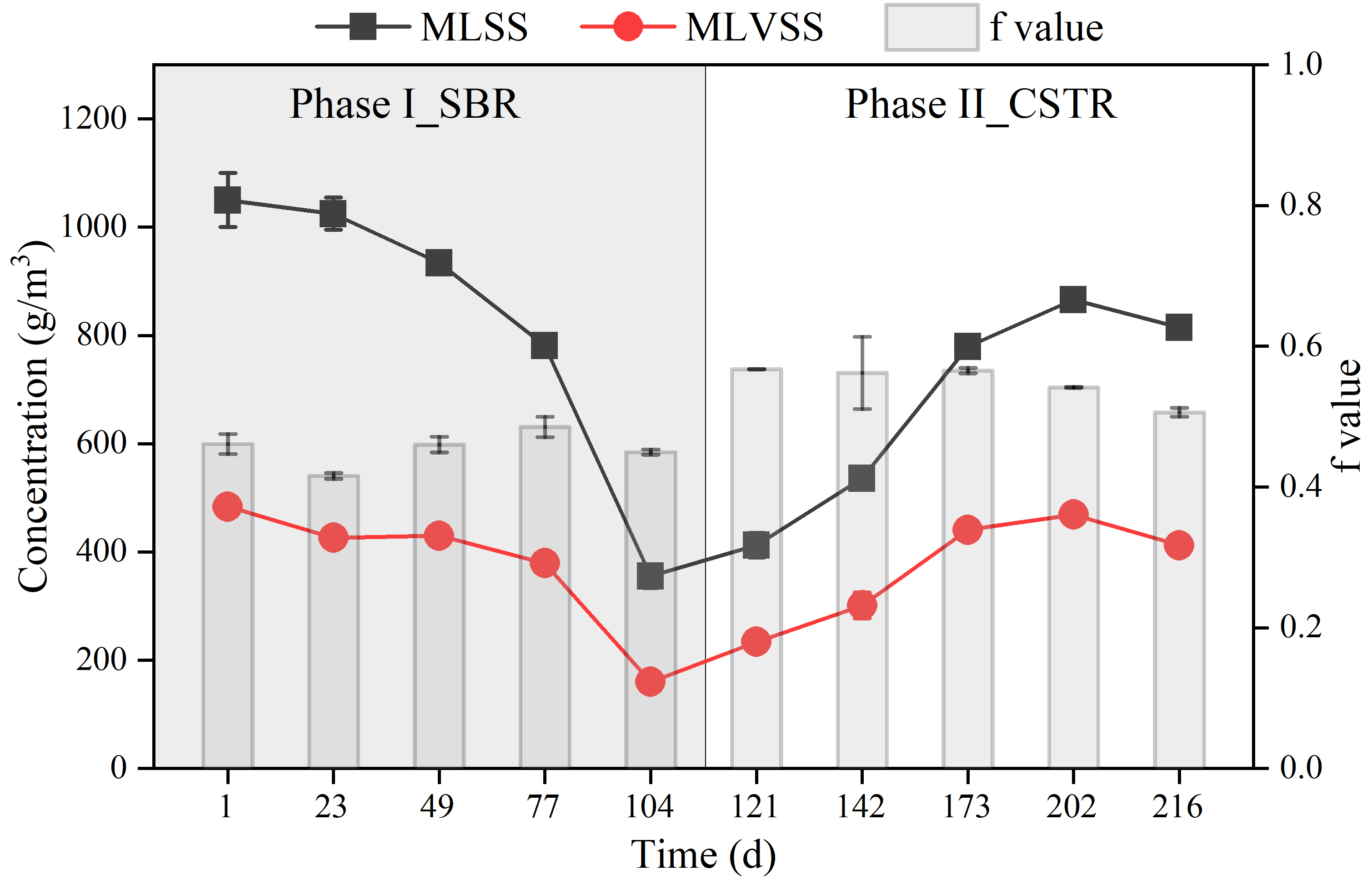


**Fig. S2.** Concentrations of MLSS and MLVSS in phase I and phase II. The f value defined as the ratio of MLVSS to MLSS was used to reflect the biological activity of floccular sludge.


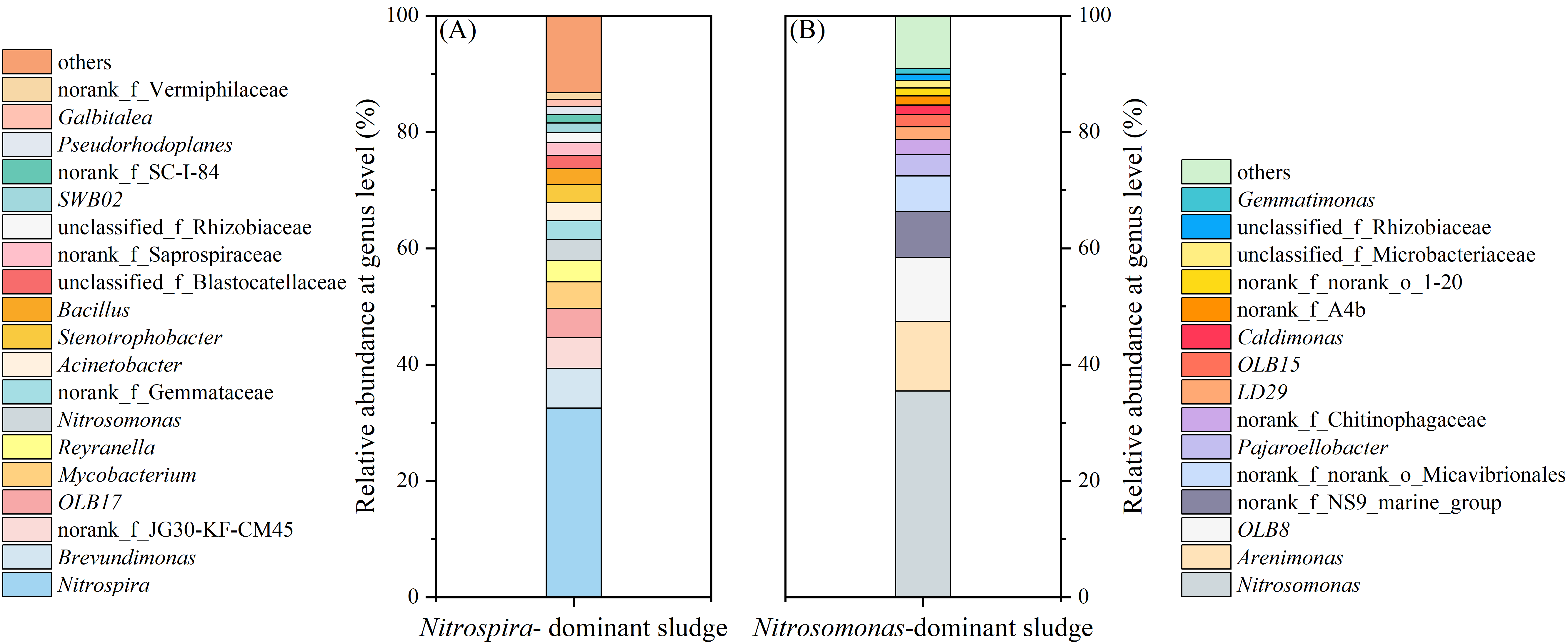
**Fig. S3.** Genera dominant in (**A**) *Nitrospira*-dominant sludge and (**B**) *Nitrosomonas*-dominant sludge.
